# Quantification of amyotrophic lateral sclerosis (ALS) disease accumulation with T1-weighted high-resolution magnetic resonance imaging: validation in an independent cohort

**DOI:** 10.1007/s00415-026-13937-4

**Published:** 2026-06-29

**Authors:** Philip Alexander Gremmler, Janina von der Gablentz, Julia Meyer, Benjamin Ilse, Behnaz Farahi Ghasraboonasr, Sophie Dalbert, Isabelle Jana Buchholz, Julian Grosskreutz, Robert Steinbach

**Affiliations:** 1 Department of Neurology, Jena University Hospital, Jena, Germany; 2 Precision Neurology of Neuromuscular and Motor Neuron Diseases, University of Lübeck, Lübeck, Germany; 3 Cluster for Precision Medicine in Inflammation, Universities of Kiel and Lübeck, Kiel, Germany; 4 MVZ Neurologie, MVZ Zentralklinik Bad Berka, Weimar, Germany

**Keywords:** Amyotrophic laterals sclerosis, D50-model, Magnetic-Resonance-Imaging (MRI), Biomarker, Voxel-Based-Morphometry (VBM), Cortical thickness

## Abstract

**Background:**

Amyotrophic Lateral Sclerosis (ALS) is a progressive neuromuscular disease with multifaceted phenotypic presentation thus obstructing objective disease staging. The D50 disease progression model is a framework to comprehensively dissect biomarker-signals towards their relevance regarding disease accumulation/phase (rD50), or disease aggressiveness (D50). Based on previous findings using 1.5-Tesla Magnetic-Resonance-Imaging (MRI), this study hypothesized that high-resolution MRI markers of Grey-Matter (GM) structural integrity would enable quantification of disease accumulation, independent of aggressiveness.

**Methods:**

A separate cohort of 75 patients with ALS and 73 Healthy Controls (HC) underwent T1-weighted 3-Tesla MRI. Voxel-Based-Morphometry measured GM and White-Matter (WM) density and Surface-Based-Morphometry assessed Cortical Thickness (CT). Non-parametric Threshold-Free-Cluster-Enhancement with 5000 permutations was applied for inter-group and regression contrasts, whilst correcting for possibly interfering co-variates and applying Family-Wise-Error-adjustment.

**Results:**

Compared with HC, the ALS cohort showed widespread decreases of CT and GM/WM density (*p* < 0.001). These case–control effects were driven by patients scanned during rD50-defined disease Phase 2 (*p* < 0.001). Within the ALS-cohort, direct Phase 2 versus Phase 1 contrasts revealed spatially-distributed decreases, reflecting higher disease accumulation (*p* < 0.05). These were independent of disease aggressiveness (and onset-region), as corrected for in the models. Accordingly, all contrasts assessing aggressiveness did not yield significant results.

**Conclusions:**

These semi-automated analyses of T1-weighted-images captured disease accumulation related GM structural integrity-loss in this cohort scanned with 3-Tesla MRI, independent of the underlying disease aggressiveness. This principle was validated across different scanners and field strengths, supporting its application for objective and non-invasive staging of patients with ALS, whereby true longitudinal studies are necessary.

**Supplementary Information:**

The online version contains supplementary material available at 10.1007/s00415-026-13937-4.

## Introduction

Amyotrophic lateral sclerosis (ALS) is a progressive neurodegenerative disorder characterized by substantial heterogeneity in clinical presentation and highly variable disease progression [1].

Due to the disease’s heterogeneity, a reliable prediction of the disease course is not possible in clinical practice. Moreover, the multifaceted phenotype and minor day to day symptom fluctuations impede objective assessment of current disease state across individuals and time [2]. Nevertheless, such an objective quantification of disease stage/accumulation is critically needed to optimize therapeutic management and to enable advanced stratification and monitoring in interventional (trial) settings in the sense of precision medicine [3]. This justifies the urgent necessity for biomarkers that can be obtained repeatedly and reproducibly in clinical practice and provide objective measures of the current disease load/accumulation.

Magnetic resonance imaging (MRI) is promising due to its non-invasive nature, broad availability, and sensitivity to subtle Grey-Matter (GM) and White-Matter (WM) alterations. In ALS, structural MRI has repeatedly shown widespread GM and WM changes in comparisons with Healthy Controls (HC) [4–7].

Nevertheless, translating imaging assessments into clinically meaningful measures remains challenging given the clinical heterogeneity and variable progression speed [8]. Imaging markers will therefore only gain reliable interpretability when embedded in clinical frameworks that separate aspects of disease accumulation from disease aggressiveness [8, 9]. Several clinical classification/staging-systems have been proposed, such as the King’s or MiToS staging [10, 11]. They usually require clinical cues or measurements and are thus potentially resource-intense and error-prone, which may partly explain why some studies showed mismatches in relation to neuroimaging [12–14].

International standard to assess current disease burden is the ALS Functional Rating Scale Revised (ALSFRS-R), a 12-item questionnaire that monitors accumulating neuromuscular dysfunctions in a semi-structured interview [15]. It has replaced survival time as the primary clinical trial outcome [16]. Main disadvantages are high inter- and intra-rater variability [2], its multidimensional construct [16–18], and nonlinear decline of sum scores over time [19–21]. To overcome the majority of these limitations, the D50 model of ALS disease progression has been developed [22, 23]. Briefly, the model describes the accumulating loss of motoric functions over time as a sigmoidal curve from full health to functional loss by iterative fitting on longitudinally assessed ALSFRS-R scorings. It quantifies overall disease aggressiveness as the time taken to reach halved functionality (D50 value) as well as relative D50 (rD50) as measure of acute disease accumulation which can be calculated for any given timepoint.

Using the D50 model, a previous MRI study scanned at a field-strength of 1.5-Tesla (1.5 T) applied Voxel-Based-Morphometry (VBM) on T1-weighted images [24]. It demonstrated a progressive spread of GM and WM pathology across rD50-derived disease Phases 1 and 2, alongside associations between VBM-WM alterations and disease accumulation. More detailed analyses of Cortical Thickness (CT) via Surface-Based-Morphometry (SBM) showed voxel-wise correlations with disease accumulation (rD50) that were independent of disease aggressiveness [25]. In contrast, WM integrity assessed via diffusion tensor imaging has shown to correlate more closely with disease aggressiveness [22]. Together, these findings suggest that in MRI GM measures derived from VBM and SBM closely reflected disease accumulation, largely independent of disease aggressiveness. In contrast, WM integrity was more strongly associated with disease aggressiveness. However, confirmation in independent cohorts is lacking, particularly using high resolution 3 T MRI within the D50 framework.

The present study therefore aimed to evaluate whether previously reported associations between structural MRI markers and clinical parameters derived from the D50 disease progression model can be replicated in an independent cohort studied with high-resolution 3 T MRI.

Based on previous findings, we hypothesized that GM integrity, assessed using CT and VBM-derived measures, primarily reflects disease accumulation (rD50), independent of overall disease aggressiveness (D50). Replication across independent cohorts and scanner field strengths would support the wide applicability of the D50 disease progression model and underscore the potential of structural MRI as a non-invasive biomarker for rD50-based disease staging in ALS. This would be an important step towards the development of MRI-based objective patient monitoring and stratification.

## Methods

### Participants

Participants were recruited at the Neuromuscular Center of Jena University Hospital (Jena, Germany) from June 2019 until June 2025. Prior to inclusion, all individuals provided written informed consent. Ethical approval was obtained from the Ethics Committee of the Friedrich Schiller University Jena (reference number 3633–11/12), and all study procedures adhered to the Declaration of Helsinki and its latest amendments.

Clinical characteristics were extracted from a local specialized neuromuscular disease database [26], including onset type (bulbar versus spinal as region of first neuromuscular weakness), age at MRI acquisition, age at diagnosis, handedness, D50 depicting overall disease aggressiveness, dx (modelling-derived time constant of longitudinal ALSFRS-R total score decline), and rD50 quantifying disease accumulation at MRI.

**Fig. 1:**
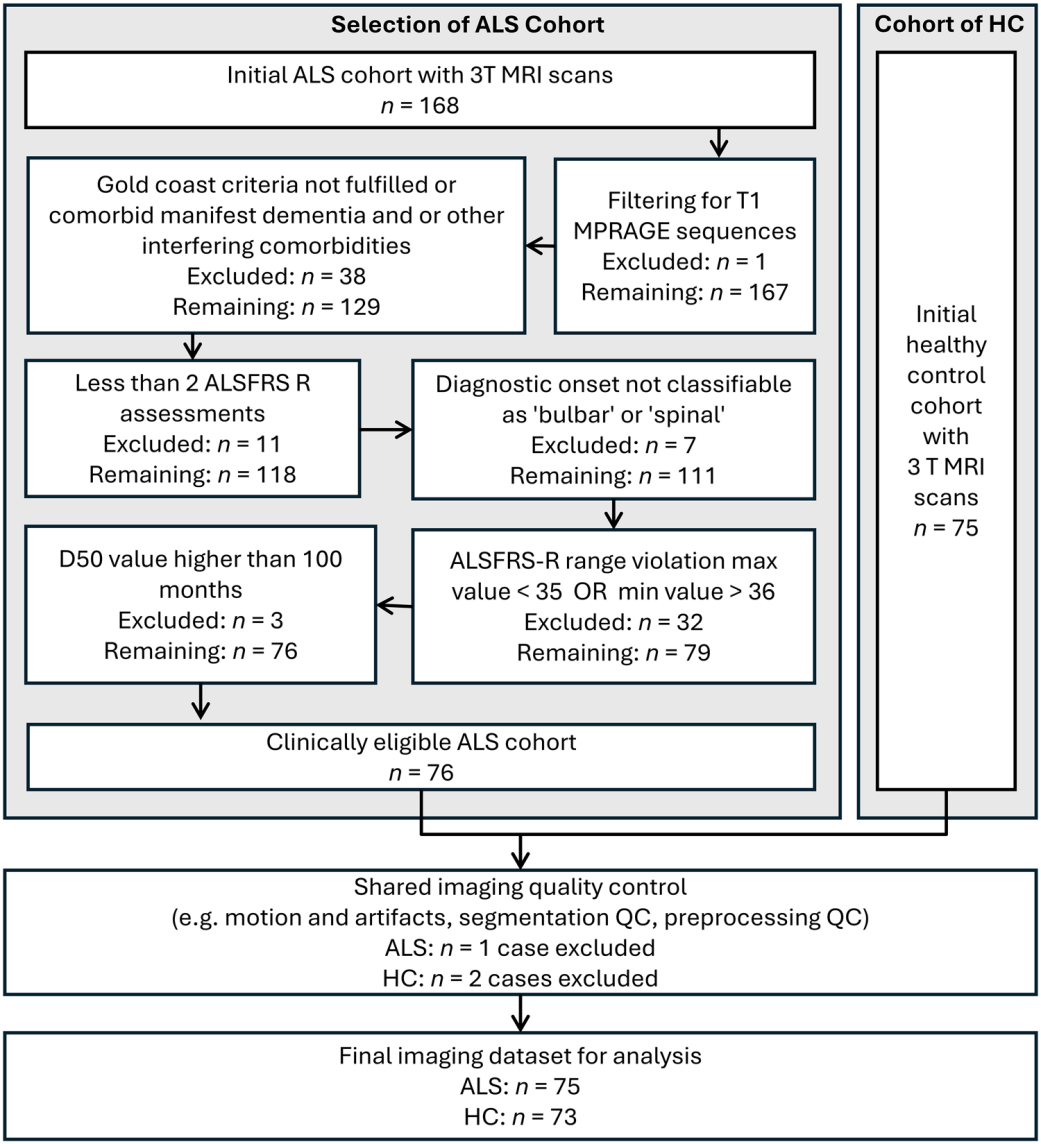
CONSORT flow-chart of participant selection for both studied cohorts. *ALS* Amyotrophic Lateral Sclerosis, *ALSFRS-R* ALS Functional Rating Scale Revised, HC Healthy Controls, *max* maximum, *min* minimum, *T* Tesla, *QC* Quality Control

A flow-chart of participant selection is presented in Fig. 1. All patients with ALS and available T1-weighted MRI scans fulfilled the Gold-Coast diagnostic criteria and could be either classified as definite, probable, or probable laboratory-supported ALS according to the revised El-Escorial criteria [27, 28]. Cases of juvenile ALS or primary lateral sclerosis were not included. Clinical exclusion criteria compromised manifest dementia symptoms or additional neurological/systemic conditions possibly confounding motor function. Patients were also excluded if the onset type could not be assigned to either the bulbar or spinal regions.

Healthy Controls (HC) were recruited from the general population and confirmed to be free of neurological or psychiatric disorders known to affect motor or cognitive performance (by careful consideration of medical history, done by RS). Their cognitive performance proved to be within normal ranges, as screened either via Mini-Mental State Examination (MMSE) [29] or a Consortium to Establish a Registry for Alzheimer's Disease (CERAD) test battery [30].

### Clinical characterization with the D50 model

The D50 disease progression model was applied for retrospective clinical characterization of the overall ALS disease course, assessing two main parameters. The D50 value represents overall disease aggressiveness, defined as the time in months until 50 percent of functionality is lost. In contrast, the relative D50 (rD50) provides a continuous (timepoint dependent) measure of disease accumulation that was calculated here for the time of MRI acquisition. To ensure stable D50 model fitting in this newly recruited cohort with partly limited follow-up, patients with ALS were selected based on criteria from recent work [23]. Patients had to have at least two consequent ALSFRS-R scorings with a maximum sum score ≥ 35 and a minimum sum score ≤ 36.

### Milestone-based clinical characterization

We retrospectively calculated King’s clinical stagings and the MiToS as available based on item-wise responses in the ALSFRS-R questionnaire using established formula [11, 31]. ALSFRS-R assessments within ± 35 days of the day of MRI acquisition were accepted (available for *n* = 62 patients). If the closest assessments before and after MRI were both outside this interval, subjects were included only when the derived King’s-stage/MiToS from the pre- and post-MRI ALSFRS-R was identical (indicating stable clinical staging around MRI). Subjects with discordant stagings before and after MRI were excluded from these analyses. We also calculated the broadly used disease progression rate (DPR; calculated as [48-ALSFRS-R]/months since symptom onset) to include it as a nuisance covariate typically attributed with disease progression speed applying equal rules: i) from an ALSFRS-R assessments within ± 35 days or ii) pre- and post-MRI calculated DPR were averaged. These results were available for 67 patients (89.3% of the cohort).

### MRI data acquisition and quality control

MRI data were acquired on a 3-Tesla (3 T) Siemens MAGNETOM Skyra scanner at Jena University Hospital using a standardized protocol including T1-weighted and FLAIR sequences with isotropic 1 × 1 × 1 mm resolutions. T1 was captured with a MPRAGE sequence using a standard Siemens head/neck 20 coil (repetition time = 2.3 ms; echo time = 0.0031 ms; flip angle = 9°; slice thickness = 1 mm; voxel size = 1 × 1 × 1 mm).

All T1-weighted raw images were visually inspected for artifacts such as blurring, ringing, or other contrast-related distortions by two experienced raters (PG and RS). In addition, the check homogeneity procedure as implemented in the Computational Anatomy Toolbox (CAT12) was applied for quality control [32].

### MRI data preprocessing

Preprocessing of the T1-weighted 3D images was conducted with Matlab2023b using the Computational Anatomy Toolbox (CAT12, version 12.9, release 2596) within the Statistical Parametric Mapping software (SPM12, version 7771 for MRI/VBM data) on a Linux Debian platform [33].

The established Voxel-Based-Morphometry (VBM) pipeline was applied with default parameters. Steps included denoising and resampling, skull stripping, segmentation into Grey-Matter (GM) and White-Matter (WM) (and cerebrospinal fluid), spatial normalization to the Montreal Neurological Institute (MNI) space, and smoothing using an isotropic Gaussian kernel of 8 mm Full-Width at Half-Maximum (FWHM).

We accordingly conducted the standard pipeline for Surface-Based-Morphometry (SBM) preprocessing, resulting in individual Cortical Thickness (CT) maps aligned with the FreeSurfer "FsAverage" template, that were spatially smoothed with a 12 mm FWHM kernel.

### Statistical analysis

Distributions of demographic and clinical variables were analysed with Matlab. Normality distribution was evaluated with the Lilliefors test [34] and these variables are reported as mean ± standard deviation. Skewed variables are reported as median ± interquartile range, and categorical variables as absolute counts and percentages. Two-sample T-Tests, Mann–Whitney U-tests, or chi-square tests were appropriately applied for group comparisons.

For matters of short description, measures derived from VBM analyses are hereon referred to as “density”.

A voxel-wise general linear model was applied on VBM-GM and -WM maps as well as to the SBM-derived CT maps. We used ANOVA designs with additional co-variates for inter-group comparisons. Contrasts including Healthy Controls (HC) were evaluated at a Family-Wise-Error (FWE) corrected threshold of *p* < 0.001 and corrected for age and gender as nuisance co-variates. Within the ALS cohort, subgroup contrasts and voxel-wise regression analyses with D50 model parameters were evaluated at *p* < 0.05 FWE-corrected. Subgroup contrasts were compared between patients in Phase 1 (early semi-stable, rD50 < 0.25) versus those in Phase 2 (early progressive, 0.25 ≤ rD50 < 0.5), as well as between patients with low disease aggressiveness (D50 ≥ 30 months) versus high aggressiveness (D50 < 30 months). In addition, voxel-wise regression analyses were conducted to assess potential correlations with the continuous parameters rD50 and D50. For all subgroup and regression analyses, onset type (bulbar vs spinal) and the respective complementary D50 model parameters were included as nuisance co-variates, as previously described [22, 35]. Specifically, rD50-defined subgroups or regressions with the parameter rD50 were adjusted for D50 and onset type, whereas contrasts assessing associations with D50 were adjusted for rD50 and onset type. All VBM contrasts were further adjusted for Total Intracranial Volume (TIV), in line with standard recommendations to control for inter-individual differences in head size.

The Threshold-Free-Cluster-Enhancement (TFCE) method was applied to all structural imaging contrasts to enable non-parametric testing (5000 permutations; TFCE toolbox, release 269) [36]. Atlas-based labeling of significant clusters was conducted using the Desikan–Killiany 40 atlas for SBM-CT and the Neuromorphometrics atlas for VBM-GM (implemented in CAT12). VBM-WM clusters were labelled using the JHU-ICBM-DTI-81 white-matter labels via SPM 12’s atlas query.

## Results

### Study cohort

The final study cohort comprised 75 patients with ALS and 73 Healthy Controls (HC). Detailed demographic and clinical characteristics are provided in Table 1, whereby distributions of gender, handedness and age did not differ between both groups. The ALS cohort was also representative of the overall ALS population treated at the tertiary centre in Jena (Table 1).

**Table 1:** Overview of demograhics of both studied cohorts (ALS and HC) and overview of disease metrics in comparison to the whole ALS cohort treated at the centre in Jena

	ALS MRI cohort	HC MRI cohort	*p*	ALS whole cohort	*p*
n	75	73		741	
Demographics					
Age at MRI [years] #	67.41 ± 15.73	69.33 ± 11.41	0.1322	–	–
	(40.91—86.16)	(50.91—85.25)			
Age at onset [years] #	66.50 ± 15.85	–	–	64 ± 14.52	0.2472
	(40.16—82.75)			(22.75—86.41)	
Gender [male/female] ⊚	41/34	35/38	0.4134	406/335	0.09836
	54.7%/45.3%	47.9%/52.1%		54.8%/45.2%	
Handedness [left/right/unknown] ⊚	2/65/8	5/68/0	[0.1913 – 0.2307]	–	–
	2.7%/86.7%/10.7%	6.8%/93.2%/0%			
Disease Metrics					
Onset type [bulbar/spinal] ⊚	33/42	–	–	254/487	0.0929
	44%/56%			34.3%/65.7%	
D50 [months] #	24.34 ± 15.65	–	–	26.87 ± 20.98	0.2310
	(8.7—85.56)			(2.6—395.2)	
dx#	9.82 ± 7.08	–	–	11.33 ± 8.83	0.2670
	(3.3—51.76)			(0.98—136.5)	
rD50 at MRI ⊞	0.25 ± 0.1	–	–	–	–
	(0.05—0.49)				
ALSFRS-R scorings near to MRI *for n* = *62*					
total score [points] ⊞	38.92 ± 5.22 (25—48)	—	—	—	—
bulbar subscore [points] #	11.00 ± 5.00 (2—12)				
cervical subscore [points] #	9 ± 5 (1—12)	—	—	—	—
lumbar subscore [points] #	9 ± 4 (4—12)				
thoracic subscore [points] #	12 ± 1 (7—12)	—	—	—	—
Disease Progression Rate near to MRI *for n* = *67* [points lost per month] #	0.67 ± 0.81 (0.00—3.00)		–	–	–
King’s Stage near to MRI *for n* = *67* [I/II/III/IVa/IVb] ⊚	I: 22 32.8% II: 27 40.3% II: 15 22.4% IVa: 2 3.0% IVb: 1 1.5%	–	–	–	–
MiToS near to MRI *for n* = *68* [0/I/II/III/IV/V] ⊚	0: 56 82.4% I: 11 16.2% II: 1 1.5% III-V: 0 0%	–	–	–	–

*ALS* Amyotrophic Lateral Sclerosis, *ALSFRS-R* ALS Functional Rating Scale Revised, *D50* disease aggressiveness, *dx* time constant of longitudinal ALSFRS-R sum score decline, *MiToS* Milano-Torino Staging system, *MRI* Magnetic-Resonance-Imaging, *rD50* relative D50 = individual disease accumulation

Continuous data are summarized for ⊞ as mean ± standard deviation and for # as median ± interquartile range (total range in brackets). For ⊚ categorial data the number of cases and percentages are given

Subgroup analyses stratified by disease phase revealed that patients in Phase 2 were significantly older, and showed higher levels of disease aggressiveness (i.e., lower D50 values) compared to patients in Phase 1 (Supplementary Table S1). This effect was previously described as ‘sampling shift’ [37]. Similarly, comparisons between patients with low versus high disease aggressiveness demonstrated differences according to rD50 (Supplementary Table S2).

### Structural MRI changes in ALS in comparison to HC

**Fig. 2:**
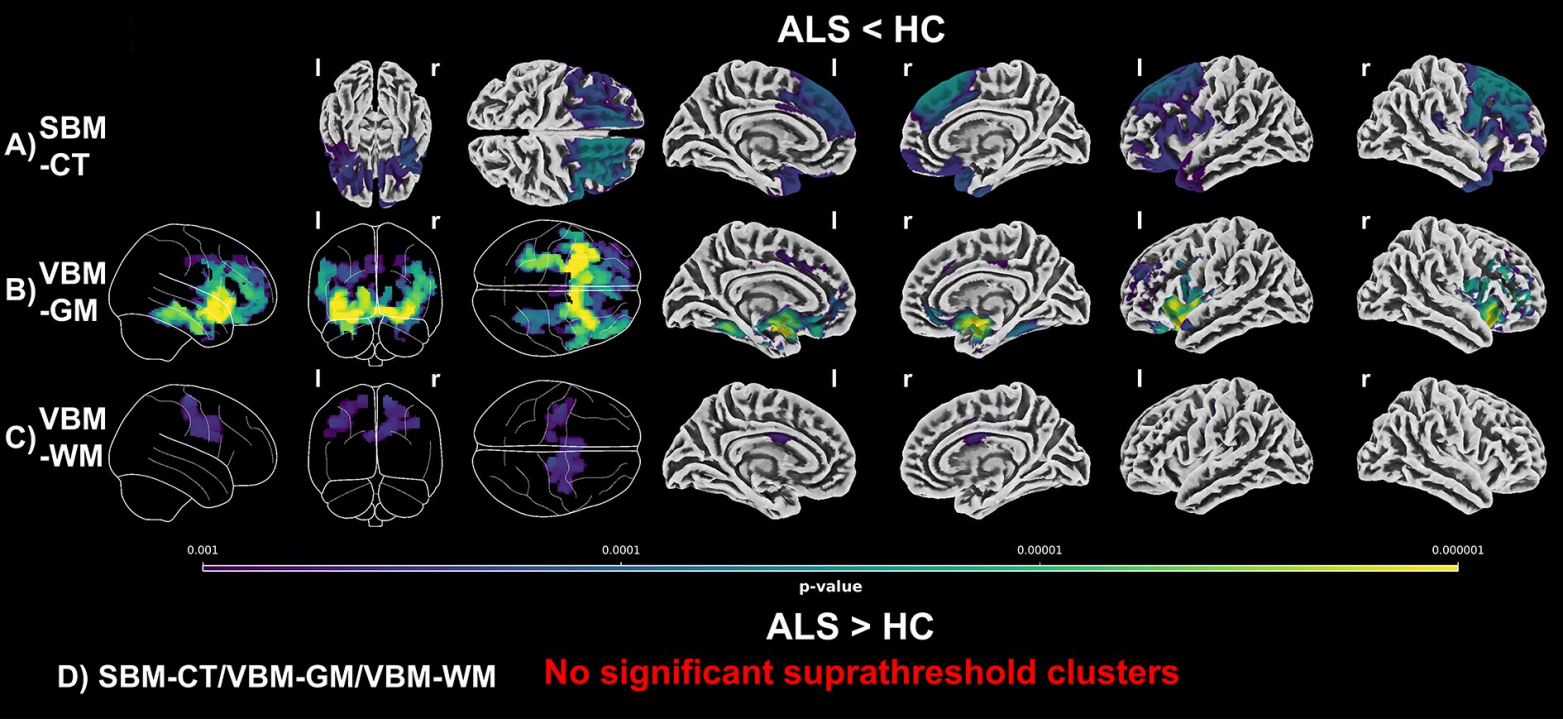
Inter-group contrasts between all patients with ALS (*n* = 75) and HC (*n* = 73). **A** SBM-CT showed bifrontal emphasized reductions for ALS (*p* < 0.001 FWE-corrected, nuisance co-variates: age and gender). **B** The VBM-GM contrast revealed overlapping but more extensive reductions in GM density (*p* < 0.001 FWE-corrected; nuisance co-variates age, gender, and TIV). **C** WM-VBM revealed bilateral ALS-related density reductions (*p* < 0.001 FWE-corrected; nuisance co-variates age, gender, and TIV). **D** None of the inverse contrasts (ALS > HC) showed any suprathreshold voxels (*p* < 0.001 FWE-corrected; nuisance co-variates age and gender, additionally TIV for VBM). *ALS* Amyotrophic Lateral Sclerosis, *CT* Cortical Thickness, *FWE* Family-Wise-Error, *GM* Grey-Matter, *HC* Healthy Controls, *l* left hemisphere, *r* right hemisphere, *SBM* Surface-Based-Morphometry, *TIV* Total Intracranial Volume, *VBM* Voxel-Based-Morphometry, *WM* White-Matter

Surface-Based-Morphometry derived Cortical Thickness (SBM-CT) was reduced in ALS patients compared with HC, mainly in frontal regions, with extension into the precentral (motor) cortex and temporo–insular areas (Fig. 2A; *p* < 0.001). This pattern was driven by superior and middle frontal cortices, including orbitofrontal and inferior-frontal subregions as well as the head/face region of the motor cortex, with a right-hemispheric emphasis. In addition, regions around the temporal pole showed decreased CT, again more evident in the right hemisphere.

Voxel-Based-Morphometry Grey-Matter (VBM-GM) showed a more widespread pattern of reduced density in ALS than suggested by the CT contrast (while overlapping in many regions; Fig. 2B; *p* < 0.001). This pattern was dominated by a large fronto–insular cluster, with additional extension into middle temporal regions.

VBM-WM density was reduced in ALS patients, centred in bilateral supratentorial WM with peak overlap in the body of the corpus callosum and extension into the right superior corona radiata (Fig. 2C; *p* < 0.001). The left-hemispheric cluster was less extensive but principally mirroring the right-sided cluster.

None of the inverse contrasts revealed any suprathreshold clusters in the sense of reduced thickness/density in HC in comparison with the ALS cohort (Fig. 2D).

### SBM and VBM changes of patients separated in disease phases versus HC

**Fig. 3:**
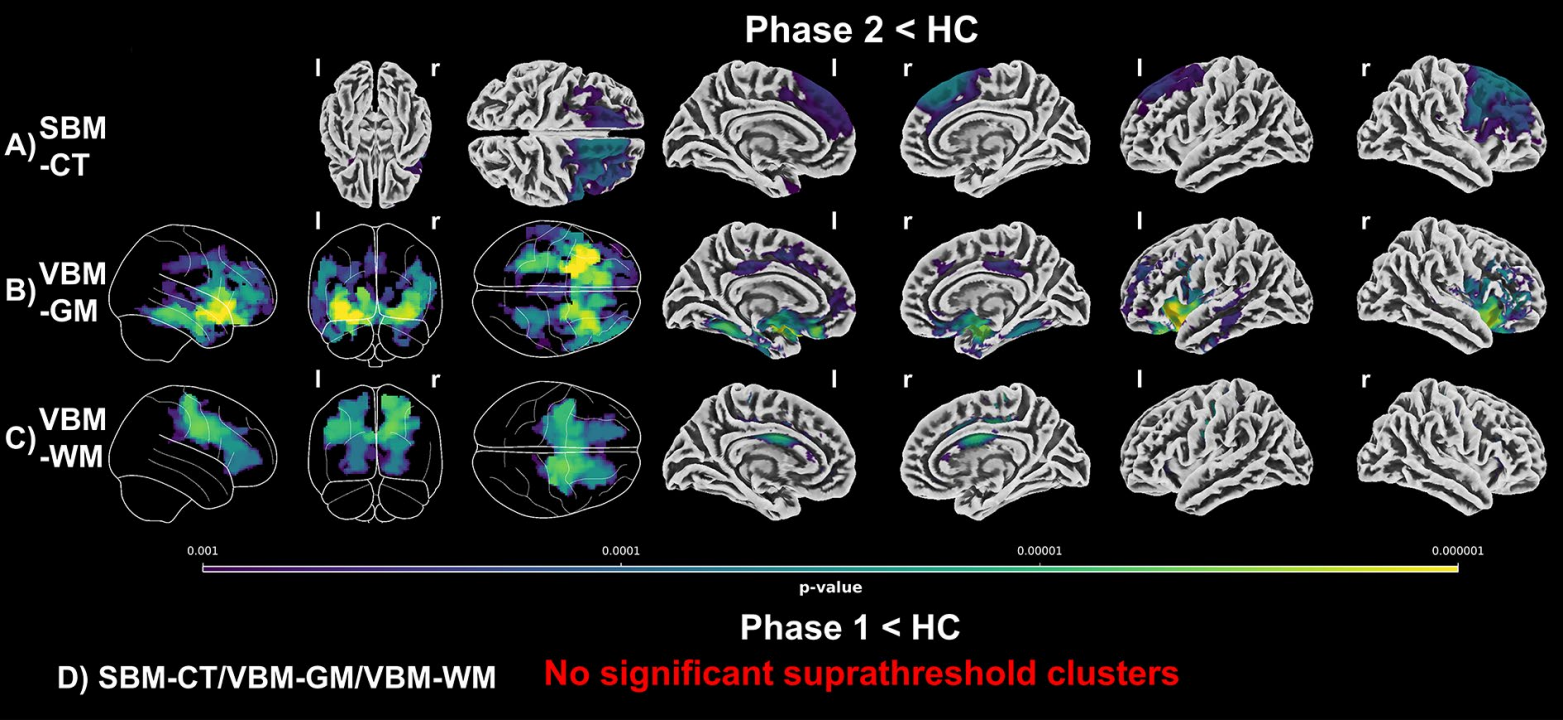
Inter-group contrasts between ALS patients separated by disease phases at the time of MRI versus HC (*n* = 73). **A** SBM-CT reductions for patients in Phase 2 (*n* = 36) versus HC (*p* < 0.001 FWE-corrected; nuisance co-variates age and gender). **B** VBM-GM density decreases of patients in Phase 2 versus HC (*p* < 0.001 FWE-corrected; nuisance co-variates age, gender and TIV). **C** VBM-WM reductions for patients in Phase 2 compared to HC (*p* < 0.001 FWE-corrected; nuisance co-variates age, gender and TIV). **D** No contrast for patients in Phase 1 (*n* = 39) versus HC showed any suprathreshold clusters (*p* < 0.001 FWE-corrected; nuisance co-variates age and gender, additionally TIV for VBM). *ALS* Amyotrophic Lateral Sclerosis, *CT* Cortical Thickness, *FWE* Family-Wise-Error, *GM* Grey-Matter, *HC* Healthy Controls, *l* left hemisphere, *MRI* Magnetic-Resonance-Imaging, *r* right hemisphere, *SBM* Surface-Based-Morphometry, *TIV* Total Intracranial Volume, *VBM* Voxel-Based-Morphometry, *WM* White-Matter

The SBM-CT contrast of patients in Phase 2 (*n* = 36) compared to HC retained the frontal signature of reductions observed in the whole cohort (versus HC), but appeared more focal, with only minor additional reductions outside frontal/motor regions (Fig. 3A; *p* < 0.001).

VBM-GM in Phase 2 (versus HC) showed a pattern of decreased density mostly comparable to that observed in the overall ALS cohort (Fig. 3B; *p* < 0.001).

VBM-WM reductions in Phase 2 compared to HC were more widespread and more bilaterally distributed than in the pooled ALS versus HC contrast under the same stringent threshold (Fig. 3C; *p* < 0.001). The Phase 2 contrast showed a large supratentorial cluster spanning the genu and body of the corpus callosum with broad bilateral extension into the corona radiata including anterior and superior divisions.

Comparisons of patients in Phase 1 (*n* = 39) towards HC did not reveal any suprathreshold clusters, neither for CT nor GM/WM contrasts at the applied threshold (Fig. 3D).

Notably, none of the inverse contrasts revealed any suprathreshold clusters, again indicating no regions with reduced thickness/density in HC compared with ALS (neither for Phase 1 nor Phase 2, not shown).

### SBM and VBM changes in relation to disease accumulation

**Fig. 4:**
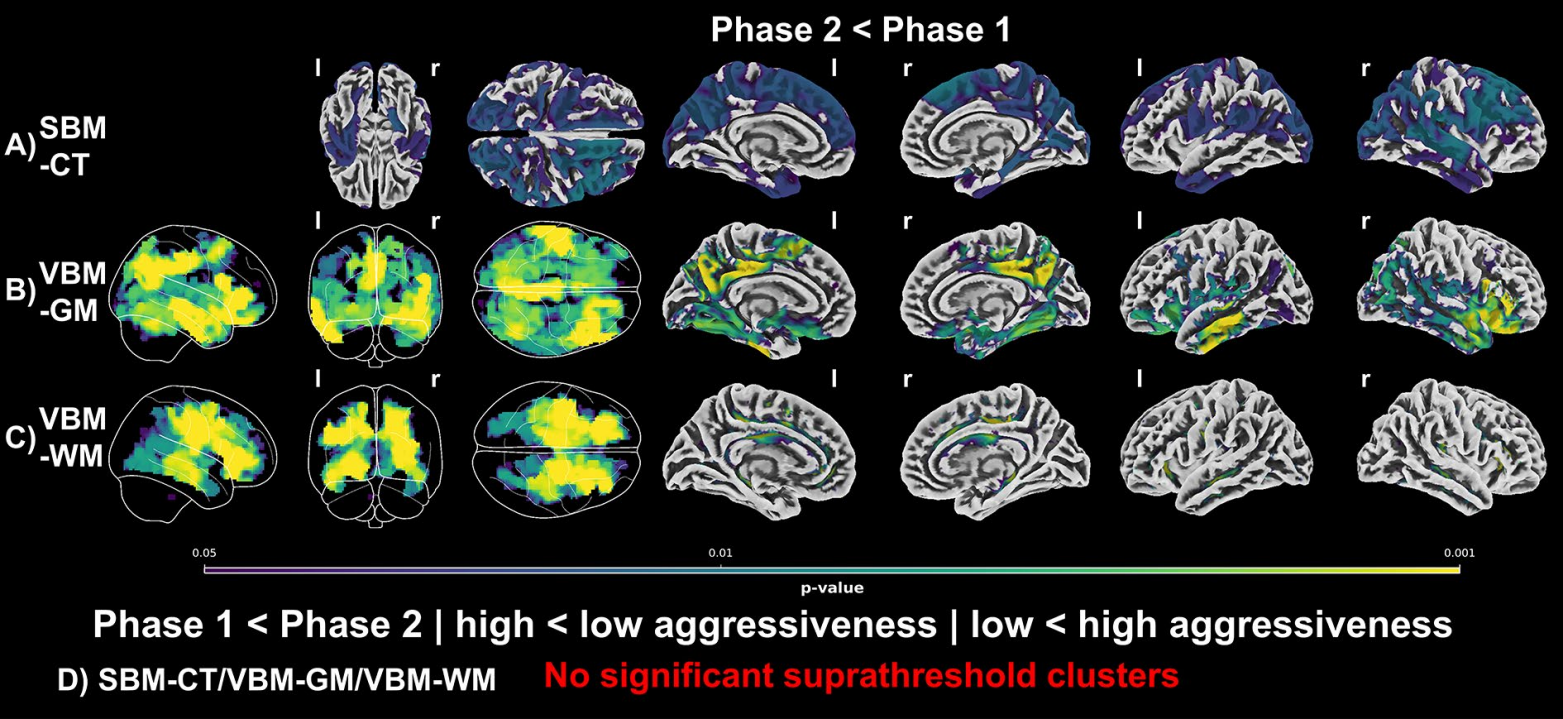
Subgroup contrasts within the ALS cohort. **A** SBM-CT for patients in Phase 2 (*n* = 36) showed reduced CT as compared to those in Phase 1 (*n* = 39) (*p* < 0.05 FWE-corrected; nuisance co-variates onset type and D50). **B** VBM-GM showed a large and spatially diffuse pattern of reduced GM density for Phase 2 patients (*p* < 0.05 FWE-corrected; nuisance co-variates onset type, D50 and TIV). **C** VBM-WM demonstrated extensive density reductions for patients in Phase 2 (*p* < 0.05 FWE-corrected; nuisance co-variates onset type, D50 and TIV). **D** None of the Phase 1 < Phase 2 and none of the contrasts comparing high aggressiveness (D50 < 30; *n* = 52) with low aggressiveness (D50 ≥ 30; *n* = 23) did reveal any suprathreshold clusters (*p* < 0.05 FWE-corrected; nuisance co-variates onset type and D50/rD50 respectively, TIV in addition for VBM). *ALS* Amyotrophic Lateral Sclerosis, *CT* Cortical Thickness, *FWE* Family-Wise-Error, *GM* Grey-Matter, *HC* Healthy Controls; *l* left hemisphere; *r* right hemisphere, *SBM* Surface-Based-Morphometry, *TIV* Total Intracranial Volume, *VBM* Voxel-Based-Morphometry, *WM* White-Matter

Within the ALS cohort, patients in Phase 2 showed a large pattern of reduced CT as compared to those in Phase 1 (Fig. 4A; *p* < 0.05). This involved bilateral fronto–parietal regions, including superior/middle frontal cortex, precentral and postcentral gyri, superior/inferior parietal cortex, and the precuneus, with additional extension into superior temporal and lateral occipital cortices.

For the same direct subgroup comparison (Phase 2 < Phase 1), VBM showed a large and spatially diffuse pattern of reduced GM density (Fig. 4B; *p* < 0.05). These effects were distributed across posterior temporal and occipito–parietal cortices, including fusiform and lingual regions, as well as the precuneus and posterior cingulate, with additional overlap in cerebellar and deep GM compartments. A separate occipital cluster (centred around the pole) indicated a posterior extension of changes with advancing disease phase.

The VBM-WM Phase 2 < Phase 1 contrast demonstrated extensive WM density reductions spanning the corpus callosum including genu, body, and posterior callosal portions and bilateral corona radiata (Fig. 4C; *p* < 0.05). Atlas based labelling further indicated cluster overlap with the posterior limb of the internal capsule and the posterior thalamic radiation. A minor cluster involved brainstem and cerebellar pathways including the medial lemniscus and inferior cerebellar peduncle.

None of the inverse contrasts (Phase 1 < Phase 2) revealed any suprathreshold clusters, indicating no regions with reduced structural integrity in patients within Phase 1 as compared to those in Phase 2 (Fig. 4D; *p* < 0.05).

Regression analyses with rD50 did not reveal any suprathreshold clusters, neither for a positive nor a negative correlation with this parameter (not shown).

### VBM and SBM changes in relation to disease aggressiveness

At the applied threshold, no significant clusters were detected for SBM-CT, VBM-GM, or VBM-WM in the subgroup comparisons of low versus high aggressiveness, nor for the inverse contrasts (Fig. 4D), nor in the positive or negative regression analyses with the parameter D50 (not shown).

### VBM and SBM changes in relation to King’s staging

For comparison purposes we also conducted milestone-based subgroup analyses applying the King’s staging, as available for 67 patients. Comparisons of patients in King’s stage 1 versus higher stages did not reveal any results in WM/GM contrasts (not shown). Subgrouping patients in King’s stage 1 and 2 together and comparing them versus those in King’s 3 or higher showed a small but primarily contra intuitive effect solely for WM-VBM (Supplementary Fig. S3). This indicated lower WM density for patients in King’s stages 1 + 2 mostly comprising the splenium of the corpus callosum.

## Discussion

This study evaluated structural T1-weighted 3 T Magnetic Resonance Imaging (MRI) correlates of Amyotrophic Lateral Sclerosis (ALS) disease course in a large cohort comprehensively characterized with the D50 model. Compared with Healthy Controls (HC), patients showed widespread Grey-Matter (GM) and White-Matter (WM) integrity loss with a fronto–temporal emphasis (Fig. 2). Subgroup stratified analyses further indicated that these differences were mainly driven by patients scanned in rD50-derived disease Phase 2 (Fig. 3). Most important, direct Phase 2 versus Phase 1 comparisons revealed extensive differences across GM and WM measures (Fig. 4). These findings support the a priori hypothesis that T1-weighted MRI reflects disease accumulation in ALS. In contrast, analyses targeting overall disease aggressiveness as defined by the D50 value, did not yield significant findings in the examined T1-contrasts.

On the case–control level, our results largely mirror findings from earlier ALS cohorts using comparable structural imaging approaches [4–6]. A previous study of 85 patients scanned with 1.5 T MRI applied VBM with TFCE and reported widespread ALS related GM and WM density decreases with a fronto-temporal emphasis [24]. The earlier work showed more extensive WM changes, which may in part reflect a higher proportion of patients in Phase 2 or beyond, as compared with the present cohort (n = 51/60% versus n = 36/48% in the current 3 T study). In the cohort of the current 3 T study, no patient had progressed beyond Phase 2 at the time of MRI. This discrepancy may reflect differences in the recruitment period and thus fewer ALSFRS-R follow-up data, as the recruitment for the older cohort studied with 1.5 T already started in 2008 (until mid-2019).

Differences may also reflect acquisition protocols (1.5 T 3D-FLASH versus 3 T MPRAGE). Although an early 1.5 T comparison reported no major differences in T1 contrast or signal-to-noise relation between MPRAGE and 3D-FLASH [38], later work showed that tissue contrast and image uniformity depend on both sequence and field strength, which can introduce regional bias patterns in tissue classification and particularly in VBM estimates [39, 40]. Thus, protocol- and field strength dependent sensitivity differences may contribute to the discrepancies observed. These issues may mainly limit cross-study comparability but do not affect the internal consistency of the present study.

CT analyses likewise showed patterns broadly comparable to those reported in a 1.5 T cohort of 100 patients with ALS [25]. In the present 3 T cohort, frontal effects were more extensive and included premotor and supplementary motor regions as well as the bulbar portion of the motor cortex, with right hemispheric emphasis (Fig. 2A). This may reflect increased sensitivity at 3 T and the use of TFCE based inference, which can improve detection of spatially distributed effects [36]. Better age-matching between the 3 T ALS cohort and HC may also contribute, whilst age and gender were still included as nuisance co-variates in both studies. In the former 1.5 T analysis this limited ALS-related effects of CT reduction associated with patients with more advanced disease accumulation/phases due to the natural intercorrelation of age and disease covered/accumulation in cross-sectional cohorts. An explanation is that patients with older age on average suffer from a faster progressing disease and are thus more impaired (higher disease accumulation) at the time of MRI scanning [22], as described before as the cross-sectional “sampling shift” [41].

A key advantage of the D50 model is that it incorporates the disease course as a whole and provides a framework to interpret cross-sectional data in a pseudo-longitudinal manner as described before [24, 41, 42]. Although this approach was already successfully applied in other studies examining biomarker signals from diverse sources, such cross-sectional results still warrant confirmation in real longitudinal studies [43–45]. Another main principle of the D50 model is that it reduces susceptibility to ALSFRS-R rating variability [2]. This framework is therefore attractive for MRI studies in ALS, since fully longitudinal imaging remains difficult due to limited feasibility, resource intensity, frequent drop-outs (e.g. due to poor longevity or interfering symptoms such as hypersalivation or orthopnoea at later stages), and selection bias (e.g., towards slow-progressors/long-survivors, younger age or spinal onset cases) [6, 46, 47].

Using relative D50 (rD50), we observed consistent structural alterations in GM and WM reflecting disease accumulation, with markedly greater impairment in Phase 2 than Phase 1 (Fig. 4). These effects presented independent of overall disease aggressiveness according to the contrasts examined in this study, as analyses of rD50 were adjusted for D50 as a nuisance covariate. Across modalities, VBM-GM showed more robust effects than CT, despite substantial regional overlap. This was also evident when applying a highly conservative Bonferroni correction to all subgroup/regression analyses, with GM-VBM changes remaining suprathreshold in inter-phase contrasts (Supplementary Fig. S1). Also, when adding age as confounder in the model, these effects were still evident, whilst CT was not (Supplementary Fig. S2). This again likely reflects the direct inter-correlation of age with the variable rD50 which diminishes the observable disease-accumulation related neuroimaging changes as observed before [25, 37]. In addition, it also sheds light on methodological differences between SBM-CT and VBM-GM when applied to ALS as a neurodegenerative disorder. Technical comparisons suggested that CT is more sensitive than VBM in normal aging, whilst the latter captures a composite GM signal influenced not only by thickness but also by surface area and folding [48]. Accordingly, VBM and SBM measures are not interchangeable and likely influenced by the underlying condition. This and former studies showed that ALS involves spatially extended multiple systems of the brain [5], whereby TFCE may further increase sensitivity [36]. In conclusion, VBM may be better suitable to detect ALS-related composite GM changes than SBM-CT [48]. Adding gender as a nuisance co-variate into the inter-phase contrasts did not relevantly change the results (data not shown). This likely mirrors that gender-distributions were not significantly different between both subgroups and trends were partly already captured by the onset-type as relatively more female patients with a bulbar onset received MRI scans during Phase 2 (see Supplementary Table S2) as typical in ALS cohorts [35, 49].

For comparison purposes we also conducted milestone-based subgroup analyses applying the King’s staging, that mainly did not reveal any results whilst only a small WM-VBM effect was revealed, indicating lower density in the anterior corpus callosum for patients in King’s stages 1 or 2 versus those in the more advanced King’s stages 3 or higher (Supplementary Fig. S3). After all, the results point towards superiority of the rD50-derived Phase allocation to reveal structural MRI changes associated with advancing disease. However, this has to be interpreted with caution as King’s staging was only retrospectively calculable for 90% of the patients and post-hoc staging may result in minor estimation errors [31]. Although King’s is per definition closer linked to clinical milestones, disadvantages for the setup of this study might be its higher degree of subdivisions (5 stages instead of 2 phases), a gap between clinically apparent affection and pre-symptomatic neurodegenerative spread to other regions or also the noise associated with the linear approximated disease progression rate included as a covariate in the contrasts [23].

We would like to emphasize, that a principal of the present study was to apply established and semi-automated whole-brain-based analysis pipelines of T1-weighted images. Based on experiences from many other neuroimaging studies in the field, this is rather unsuited to detect changes in smaller extra-neocortical regions of the brain. An example is the infratentorial cerebellum that despite partly demonstrated involvement in inter-phase contrasts (Fig. 3B; Fig. 4B,C) usually demands specific analysis pipelines given its more fine-grained anatomy requiring specialized templates/maps [50]. Applying such approaches, cerebellar pathology was increasingly recognized as part of the multisystem involvement in ALS before [51–53]. Similarly, changes of deep grey-matter such as basal ganglia were more likely detected with dedicated pipelines [25, 54].

In addition, the given cross-sectional study used retrospectively assigned disease accumulation/aggressiveness parameters based on the clinical evolution of neuromotor function-loss for subgroup/regression analyses of neuroimaging-derived measures. As opposed to this, recent studies used imaging-guided approaches with clustering to untangle disease heterogeneity in ALS and thus successfully identified 2–3 spatial sub-phenotypes of the ALS disease spectrum [12, 55]. Within this context and beyond, machine-learning represents a promising methodology to interpret MRI as a categorization tool on a group level. However, major challenges of machine learning such as high-dimensionality and co-linearity of input neuroimaging data, over-fitting, or hallucinations lead to interpretation ambiguity of derived results which is why validations in direct MRI-to-clinic association studies are still required. Therefore, the present study may present a valid starting point for the development of viable computational models to interpret single imaging datasets as individual non-invasive classification tools, that are urgently needed for a variety of clinical and clinical trial applications [56, 57].

In contrast to the rather consistent results of this study showing decreasing structural integrity in association with increasing disease accumulation, subgroup analyses targeting disease aggressiveness and regression analyses with D50 did not reveal significant findings, consistent with earlier SBM-CT work on 1.5 T scans [25]. This former study however reported a negative correlation of CT with rD50, which we could not replicate. It remains unclear whether this difference reflects cohort characteristics such as sample size (*n* = 100 versus *n* = 75) or phase distribution, or methodological and acquisition related differences. Notably, cognitive screening within normal range was used as a selection criterion in the former 1.5 T cohort, whereas such information was not entirely available here [25]. Given that higher degrees of cognitive impairment are on a cohort-level usually attributed with faster disease progressiveness in ALS, future studies incorporating cognitive and behavioural profiling may improve sensitivity for detecting structural correlates of rD50-related neuromuscular disease load [58, 59].

Notwithstanding, the absence of encompassing cognitive and behavioural profiling remains a limitation of our study, although efforts were undertaken to at least screen all our patients with the ECAS [60, 61]. Nevertheless, assessing these symptoms remains a challenge, especially for behavioural changes that rely on carer-interviews [62]. In our cohort coverage gaps were to a certain degree also related to restrictions associated with the early COVID-19 pandemic fully affecting the recruitment-period of this study [26]. Another limitation are the missing Diffusion-Tensor-Imaging analyses, which were not comparatively available due to a scanner software related change in the specific protocol (not affecting the rest of the protocol, e.g., T1). Prior diffusion MRI studies of WM regions have suggested that disease aggressiveness is more closely linked to derived alterations in main fibre bundles [22]. Future, combined analyses will therefore help to decipher these effects in 3 T imaging of WM. As a further limitation, genetic profiles were also not available for the entire ALS cohort but would be desirable to search for potential associations of our neuroimaging findings with for example presence of C9orf72 expansions [63, 64] or UCN13A [65]. However, conflicting results in an extensive cohort recently suggested that patients with ALS-related variants in the same genetic regions may rather show the entire neurodegenerative spectrum as detectable with MRI-clustering [55]. This underlines that genotype-to-neuroimaging association studies probably need a much larger data-basis with more in-depth genetic characterization in future research [66, 67].

In conclusion, this study revealed that T1-weighted MRI is capable of detecting loss of structural integrity mirroring disease accumulation especially in GM, whilst these changes presented independent of the underlying disease aggressiveness thus matching the a-priori hypothesis. As this principal is confirmed across scanners and field strengths in cross-sectional studies, it supports the rational to use T1 MRI as a non-invasive objective monitoring tool, in observational and interventional trial settings. Nevertheless, confirmation through real-longitudinal studies is required [9, 68].

## Supplementary Information

Below is the link to the electronic supplementary material.

Supplementary file1 (DOCX 1491 KB)

## Data Availability

The deidentified data supporting the findings of this study are available from the corresponding author upon reasonable request.

## Abbreviations

ALS: Amyotrophic Lateral Sclerosis
MRI: Magnetic Resonance Imaging
FWE: Family-Wise-Error
GM: Grey-Matter
WM: White-Matter
HC: Healthy Controls
ALSFRS-R: ALS Functional Rating Scale Revised
rD50: Relative D50 (individual disease accumulation/covered)
VBM: Voxel-Based-Morphometry
CT: Cortical Thickness
SBM: Surface-Based-Morphometry
T: Tesla
TFCE: Threshold-Free-Cluster-Enhancement
CAT12: Computational Anatomy Toolbox
