# Supplementary material for: Quantification of amyotrophic lateral sclerosis (ALS) disease accumulation with T1-weighted high-resolution magnetic resonance imaging: validation in an independent cohort
Source: J Neurol. 2026 Jun 29;273(7):429. doi: 10.1007/s00415-026-13937-4 (PMC13314698; doi:10.1007/s00415-026-13937-4)
Supplement: Supplementary file 1 — Supplementary file1 (DOCX 1491 KB) [file 415_2026_13937_MOESM1_ESM.docx]

# **Supplementary Information for article:**

**Quantification of Amyotrophic Lateral Sclerosis (ALS) disease accumulation with T1-weighted high-resolution Magnetic Resonance Imaging: validation in an independent cohort**

Corresponding author:

Robert Steinbach

Scientific head of the Center for Neuromuscular Diseases Jena

Neurologist at Jena University Hospital

Am Klinikum 1, 07747 Jena, Germany

E-mail: Robert.Steinbach@med.uni-jena.de

**Supplementary Figure 1**


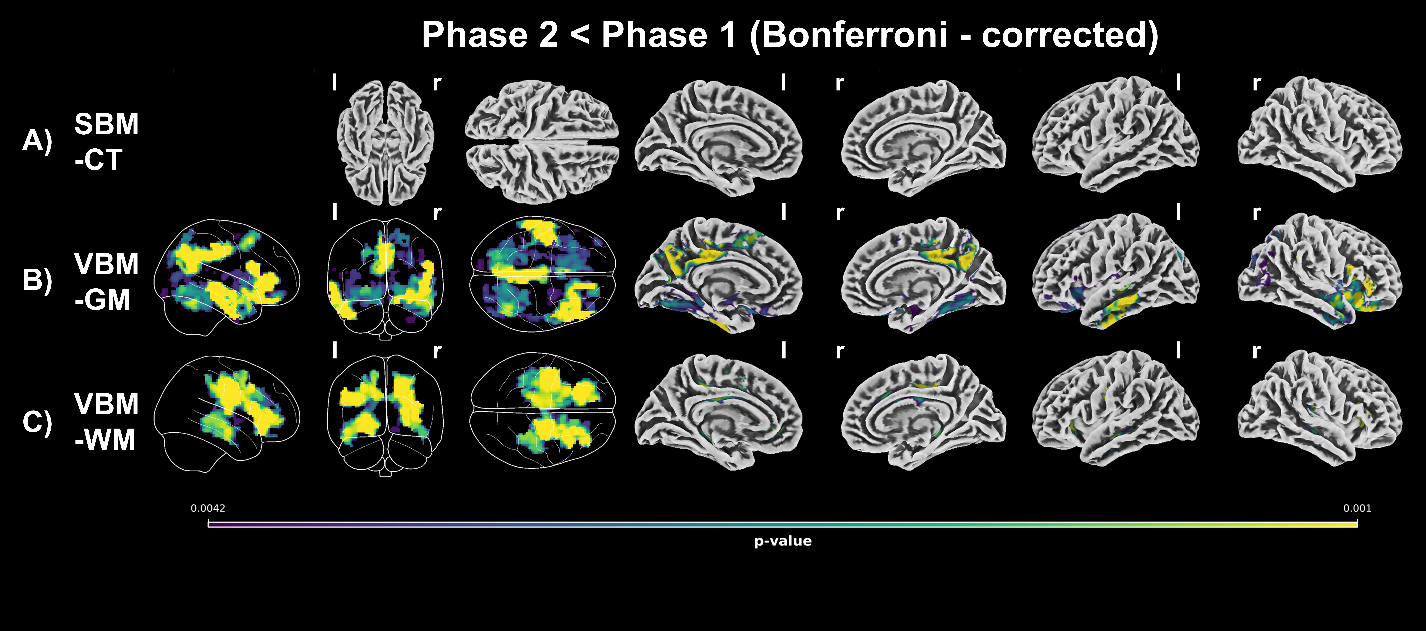


Phase 2 < Phase 1 after Bonferroni correctiton. SBM: p<0.0042 FWE-corrected, nusiance co-variates: onset-type, D50. VBM: p<0.0042 FWE-corrected, nusiance co-variates: onset-type, D50, TIV.

**Supplementary Figure 2**


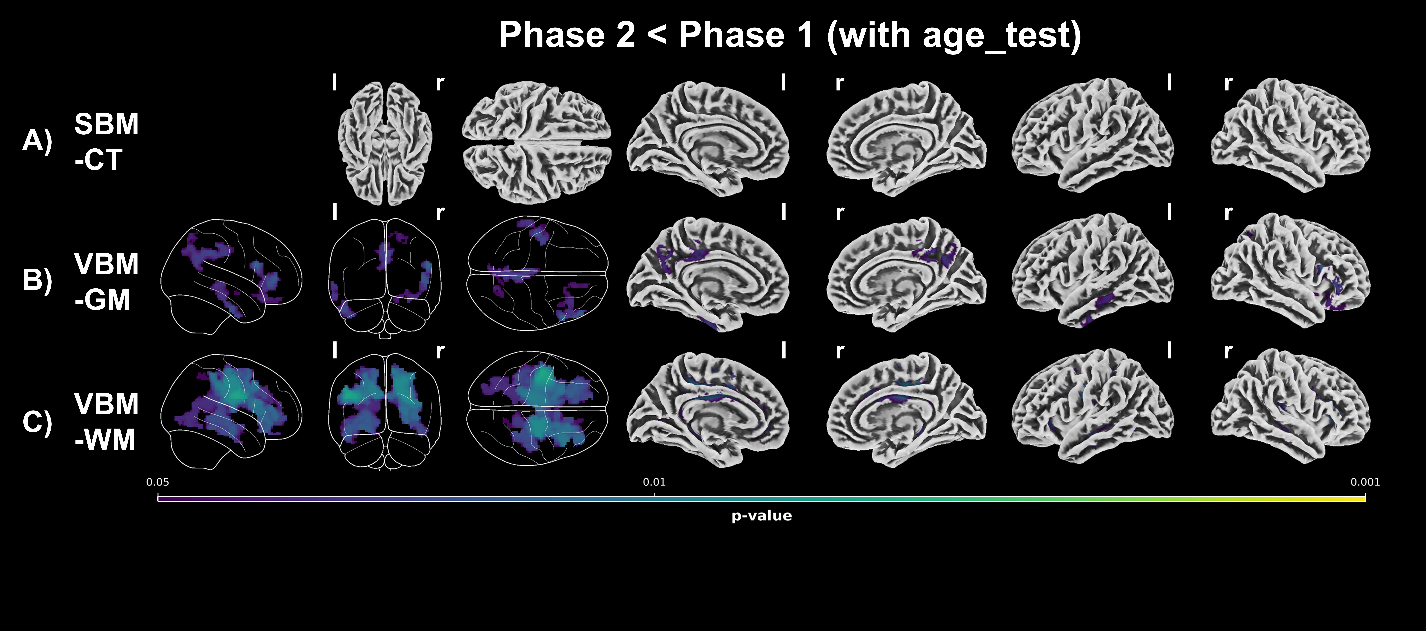


Phase 2 < Phase 1 with age as additional nuisance covariate. SBM: p<0.05 FWE-corrected, nusiance co-variates: age, onset-type, D50. VBM: p<0.05 FWE-corrected, nusiance co-variates:age, onset-type, D50, TIV

**Supplementary Figure 3**

**
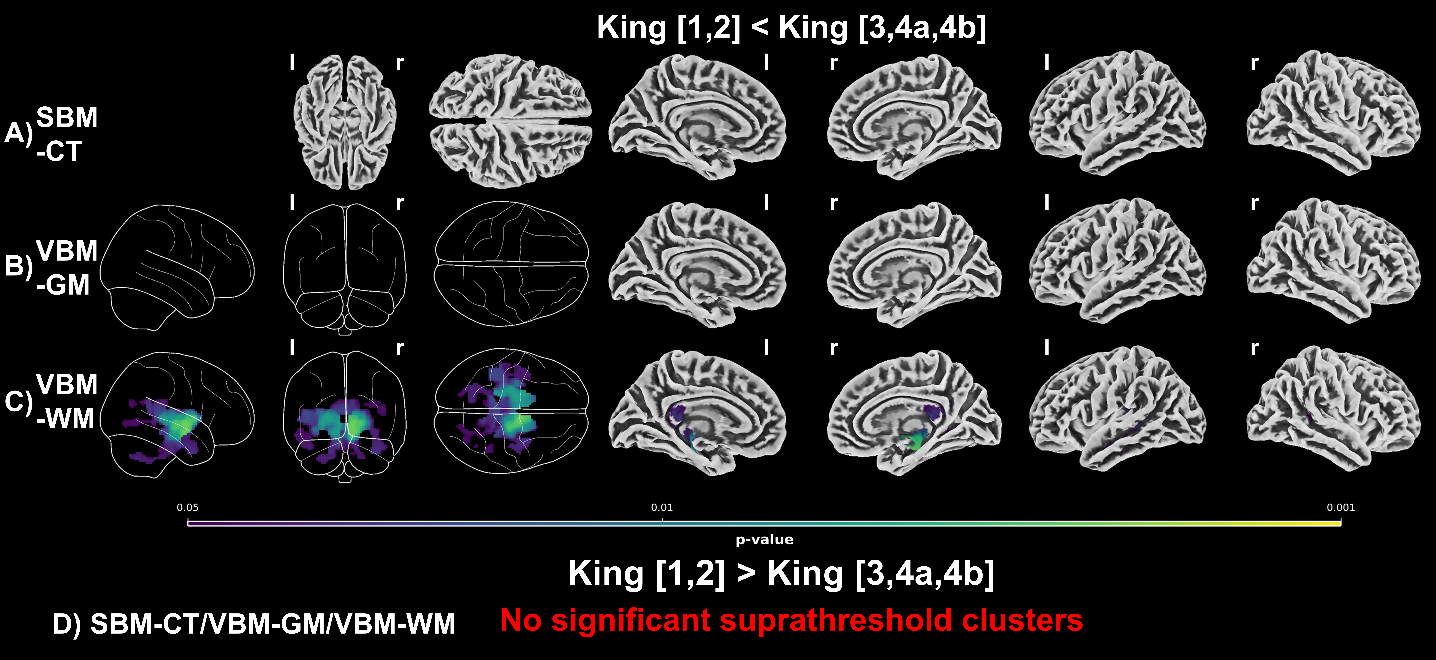
**

Kingstage [1,2] < Kingstage [3,4,4a,4b] with Disease Progression rate (DPR) as additional nuisance covariate. SBM: p<0.05 FWE-corrected, nusiance co-variates: DPR , onset-type, VBM: p<0.05 FWE-corrected, nusiance co-variates: DPR, onset-type, TIV.

**Supplementary Table 1:**

| ALS (*n* = 75) | Phase 1  (rD50 < 0.25) | Phase 2  (rD50 >=0.25) | *p* |
| --- | --- | --- | --- |
| n | 39  52% | 36  48% |  |
| *Demographics*  Age at MRI [years] # | 63.91 ± 17.69  (40.91 - 77.83) | 70.08 ± 11.34  (48.08 - 86.16) | ***0.0152**** |
| Gender [male/female] ⊚ | 25/14  64.1% / 35.9% | 16/20  44.4% / 55.6% | *0.0875* |
| *Disease Metrics*  Onset type [bulbar/spinal] ⊚ | 14/25  35.9% / 64.1% | 19/17  52.8%/47.2% | *0.1412* |
| ALSFRS-R scorings near to MRI *for n=62*  total score [points] ⊞ | 41.81±3.58  (34.00 - 48.00) | 36.03 ± 5.04  (25.00 - 44.00) | *<0.0001* |
| bulbar Subscore near to MRI *for n=62* [points] # | 11.00 ± 2.00  (6.00 - 12.00) | 9.00 ± 6.75  (2.00 - 12.00) | *0.0047* |
| cervical Subscore near to MRI *for n=62* [points] # | 10.00 ± 3.00  (5.00 - 12.00) | 8.00 ± 5.50  (1.00 - 12.00) | *0.0159* |
| lumbar Subscore near to MRI *for n=62* [points] ⊞ | 9.68 ± 2.12  (5.00 - 12.00) | 8.77 ± 2.32  (4.00 - 12.00) | *0.1147* |
| thoracic Subscore near to MRI *for n=62* [points] # | 12.00 ± 0.00  (9.00 - 12.00) | 12.00 ± 1.00  (7.00 - 12.00) | *0.2749* |
| Disease Progression Rate near to MRI *for n=67* [points lost per month] # | 0.50 ± 0.58  (0.00 – 3.00) | 0.78 ± 0.99  (0.1 – 2.57) | *0.0835* |
| King’s Stage near to MRI *for n=67* [0/I/II/III/IVa/IVb] ⊚ | I: 15 (45.5%)  II: 12 (36.4%)  III: 6 (18.2%)  IVa: 0 (0.0%)  IVb: 0 (0.0%) | I: 7 (20.6%)  II: 15 (44.1%)  III: 9 (26.5%)  IVa: 2 (5.9%)  IVb: 1 (2.9%) | *0.1452* |
| MiToS near to MRI *for n=68*  [0/I/II/III/IV/V] ⊚ | 0: 32 (91.4%)  I: 3 (8.6%)  II: 0 (0%)  III-V: 0 (0%) | 0: 24 (72.7%)  I: 8 (24.2%)  II: 1 (3%)  III-V: 0 (0%) | *0.0431* |
| Relative D50 [months] # | 0.18 ± 0.07  (0.05 - 0.24) | 0.32 ± 0.10  (0.26 - 0.49) | *-* |
| D50 [months] # | 28.09 ± 19.87  (13.30 - 85.56) | 20.73 ± 14.68  (8.70 - 82.65) | ***0.0073**** |
| dx# | 12.08 ± 8.78  (6.18 - 51.76) | 8.34 ± 6.24  (3.30 - 33.24) | ***0.0010**** |
| Continuous data are summarized for ⊞ as mean ± standard deviation and for # as median ± interquartile range (each with the total range in brackets). For ⊚ categorial data. the number of cases and percentages are given. Variables that are time-point dependent refer to the day of MRI-acquisition; others depict constant characterization of patients’ overall disease course.  *Abbreviations*: *D50* estimated time in months for an individual to lose 50% of functionality; *dx* time constant of longitudinal ALSFRS-R total score decline; *MRI* Magnetic Resonance Imaging; *rD50* (relative D50) individual disease accumulation. | | | |

**Supplementary Table 2:**

| ALS (*n* = 75) | Low Aggressiveness (D50 ≥ 30 months) | High Aggressiveness (D50 < 30 months) | *p* |
| --- | --- | --- | --- |
| n | 23  30.66% | 52  69.33% |  |
| *Demographics*  Age at MRI [years] # | 66.83 ± 10.81  (44.33 - 86.16) | 69.91 ± 15.96  (40.91 - 82.66) | *0.2553* |
| Gender [male/female] ⊚ | 13 / 10  56.5% / 43.5% | 28 / 24  53.8% /46.2% | *0.8301* |
| *Disease Metrics*  Onset type [bulbar/spinal] ⊚ | 7/16  30.4%/69.6% | 26/26  50%/50% | *0.1155* |
| ALSFRS-R scorings near to MRI *for n=62*  total score [points] ⊞ | 40.63 ± 4.19  (30 - 47) | 38.16 ± 5.49  (25 - 48) | *0.0863* |
| bulbar Subscore near to MRI *for n=62* [points] # | 12.00 ± 2.75  (4 - 12) | 10.00 ± 5.75  (2 - 12) | *0.0485* |
| cervical Subscore near to MRI *for n=62* [points] # | 10.00 ± 2.00  (4 - 12) | 9.00 ± 5.75  (1 - 12) | 0.4958 |
| lumbar Subscore near to MRI *for n=62* [points] # | 9.00 ± 2.75  (5 - 12) | 10.00 ± 4.00  (4 - 12) | *0.5612* |
| thoracic Subscore near to MRI *for n=62* [points] # | 12.00 ± 0.75  (9 - 12) | 12.00 ± 1.00  (7 - 12) | *0.7965* |
| Disease Progression Rate near to MRI *for n=67* [points lost per month] # | 0.40 ± 0.22  (0.1 - 1.21) | 0.85 ± 0.90  (0.00 - 3.00) | *<0.0001* |
| King’s Stage near to MRI *for n=67* [0/I/II/III/IVa/IVb] ⊚ | I: 7 (31.8%)  II: 10 (45.5%)  III: 4 (18.2%)  IVa: 1 (4.5%)  IVb: 0 (0.0%) | I: 15 (33.3%)  II: 17 (37.8%)  III: 11 (24.4%)  IVa: 1 (2.2%)  IVb: 1 (2.2%) | *0.8712* |
| MiToS near to MRI *for n=68*  [0/I/II/III/IV/V] ⊚ | 0: 22 (95.7%)  I: 1 (4.3%)  II: 0 (0 %)  III-V: 0 (0%) | 0: 34 (75.6 %)  I: 10 (22.2%)  II: 1 (2.2%)  III-V: 0 (0%) | *0.1186* |
| Relative D50[months] ⊞ | 0.20 ± 0.10  (0.06 - 0.4) | 0.27 ± 0.10  (0.05 - 0.49) | ***0.0071**** |
| D50 [months] # | 41.68 ± 25.55  (32.13 - 85.56) | 20.07 ± 10.50  (8.7 - 29.85) | *-* |
| dx ⊞ | 22.43 ± 9.62  (11.84 - 51.76) | 8.36 ± 2.79  (3.3 - 14.84) | *<0.0001* |
| Continuous data are summarized for ⊞ as mean ± standard deviation and for # as median ± interquartile range (each with the total range in brackets). For ⊚ categorial data. the number of cases and percentages are given. Variables that are time-point dependent refer to the day of MRI-acquisition; others depict constant characterization of patients’ overall disease course.  *Abbreviations*: *D50* estimated time in months for an individual to lose 50% of functionality; *dx* time constant of longitudinal ALSFRS-R total score decline; *MRI* Magnetic Resonance Imaging; *rD50* (relative D50) individual disease accumulation. | | | |
